# Acetylation of Never-Dried Wood via Diffusion-Driven Solvent Exchange

**DOI:** 10.1021/acs.biomac.6c00768

**Published:** 2026-06-11

**Authors:** Md Tipu Sultan, Mikko Valkonen, Muhammad Awais, Paula Nousiainen, Kristiina Lillqvist, Shennan Wang, Lauri Rautkari

**Affiliations:** Department of Bioproducts and Biosystems, School of Chemical Engineering, 174277 Aalto University, Espoo FI-02150, Finland

## Abstract

Acetylation is one of the most widely applied chemical modification methods for enhancing the dimensional stability and durability of wood. In this work, we investigate a diffusion-driven solvent-exchange method for acetylating never-dried Scots pine wood, eliminating the energy-intensive drying typically required before wood acetylation. Results from two-dimensional confocal Raman microspectroscopic imaging, coupled with multivariate data analysis and high-performance liquid chromatography, suggest a uniform distribution of acetyl groups within the cell walls and increased lignin acetylation using the solvent-exchange method. The uniform distribution of acetyl groups and increased lignin modification in the cell corners and the middle lamella resulted in less shrinkage and much-improved antiswelling efficiency at the macroscale. Overall, this diffusion-driven solvent-exchange pretreatment provides a uniform, direct acetylation of never-dried wood, bypassing the conventional oven- or kiln-drying step, with targeted lignin modification playing a crucial role in controlling wood-water interactions.

## Graphical abstract

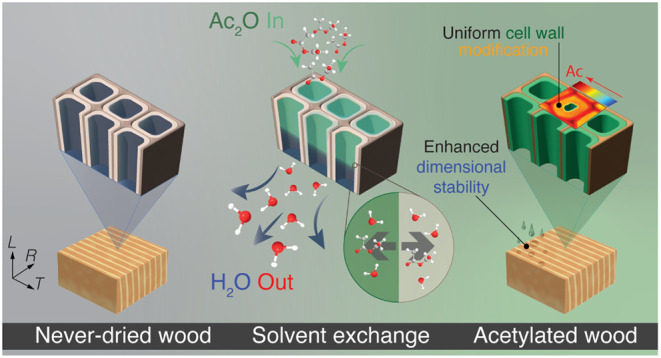

## Introduction

Native wood is susceptible to swelling, shrinkage, and fungal decay due to water sorption and desorption associated with the hydroxyl (−OH) groups in its principal cell wall polymers (cellulose, hemicelluloses, and lignin).^1,2^ Covalent chemical modification is commonly used to address these problems by substituting hydrophilic −OH groups with hydrophobic groups or moieties, thereby decreasing the number of accessible sorption sites for water and improving dimensional stability and resistance to decay.^3,4^ Acetylation is by far one of the most efficient and effective commercial chemical modification methods for wood. The covalently introduced acetyl [−C­(=O)–CH_3_] groups can effectively dehydrate the cell wall polymer network and limit water diffusion into the wood cell wall.^5^ Wood acetylation occurs via esterification between accessible −OH groups in wood and acetic anhydride (Ac_2_O). Due to the exothermic reaction of Ac_2_O with water, which is often accelerated by the released heat and catalyzed by the reaction product acetic acid (AcOH), acetylation is typically performed under anhydrous or near-anhydrous conditions, often requiring prior drying of wood by air or kiln drying. Drying also removes the reagent-flow-resisting capillary free water, allowing pressure-assisted impregnation of chemicals. However, drying wood is an energy-intensive process, accounting for 50–70% of the total energy used in wood processing.^6^ The need to dry the wood before impregnating it and reacting its available functional groups can be viewed as an additional, unnecessary step from an energy-use standpoint.

In addition to the associated energy consumption, drying also significantly affects the cell wall microstructure and molecular structure. In an earlier study,^7^ wood dried in a conventional oven at 105 °C exhibited a low specific surface area (SSA) of 0.5 m^2^ g^–1^ and mesopore (2–50 nm) volume *V*_meso_ of 0.002 cm^3^ g^–1^. In contrast, drying wood from toluene through a solvent-exchange process retained a much more open structure (SSA: 6 m^2^ g^–1^ and *V*_meso_: 0.015 cm^3^ g^–1^).^7^ Nevertheless, the mesoporosity of wood in the dry state is far less than in the fully swollen state (SSA: 198 m^2^ g^–1^ and *V*_meso_: 0.25 cm^3^ g^–1^).^8^ Beyond mesoporosity, drying has also been identified to influence cell wall microporosity (pore size < 2 nm).^9^ Nopens et al.^9^ investigated the cell wall porosity of dry and wet wood using gas physisorption and thermoporosimetry. They suggested that micropores may facilitate water transport in the cell wall.^9^ The micropore volume in dried wood was also found to be inversely correlated with increasing temperature from 20 to 180 °C, or with repeated dry-wet cycles in nature over time scales of 0.1 to 1000 years.^10,11^ Drying not only alters the cell wall porosity but also induces molecular-level structural changes. Kyyrö et al.^12^ reported the −OH accessibility to water vapor in never-dried and once oven-dried Norway spruce [*Picea abies* (L.) H. Karst.] to be 9.5 and 9.3 mmol g^–1^, respectively. Engelund Thybring et al.^13^ similarly reported a 3% decrease in relative −OH accessibility to liquid water after oven-drying of never-dried wood. Consequently, drying at high temperatures and for prolonged periods leads to molecular rearrangement of cell wall components, collapses the meso- and micropores, reduces pathways for chemical diffusion, and blocks accessible hydroxyl groups available for acetylation.^14,15^

Earlier studies have confirmed that preswollen wood reacts more readily than dried wood, underscoring the importance of maintaining an open cell wall structure during chemical treatments. For example, Hill et al.^16^ performed acetylation and propionylation of Scots pine (*P. sylvestris* L.) and Corsican pine (*P. nigra*). They found that reactions occurred mainly when wood was preswollen, produced by solvent exchange followed by evaporative drying.^16^ Obataya and Minato^17^ further showed that preswelling improves the initial acetylation efficiency by facilitating reagent diffusion into wood polymers, where hydrogen bonds had already been partially disrupted. They achieved this state through a gradual solvent exchange from water to methanol, then to acetone, and finally to Ac_2_O, carefully controlling the process to prevent premature reaction.^17^ In agreement with these results, Jakes et al.^18^ also mentioned that moisture reduces the glass-transition temperature of various polymers in wood, enhancing chemical diffusion within the wood polymer. However, the aforementioned preswelling or solvent-exchange processes in the earlier studies were applied to dry wood, in which irreversible cell wall structural changes had already been induced by oven-drying, thereby impairing the effectiveness of reswelling via solvent exchange.^19^

In this work, we explore a diffusion-driven solvent exchange process as a pretreatment for never-dried Scots pine wood to avoid the conventional predrying before acetylation and to achieve acetylation of wood in a preswollen state. Maintaining the open-pore structure of never-dried wood via solvent exchange should promote deeper, more uniform penetration of Ac_2_O, providing a potentially more effective and thorough pathway for wood acetylation. Hence, this study aims to determine the effect of solvent exchange on the molecular degree of modification, the microscopic distribution of acetylation, and the macroscopic dimensional stability of acetylated wood compared with oven-drying. Moreover, a direct comparative acetylation is performed from the never-dried state to better understand the mechanism of the solvent exchange. The acetylation strategies used are listed in Figure 1.

**Figure 1:**
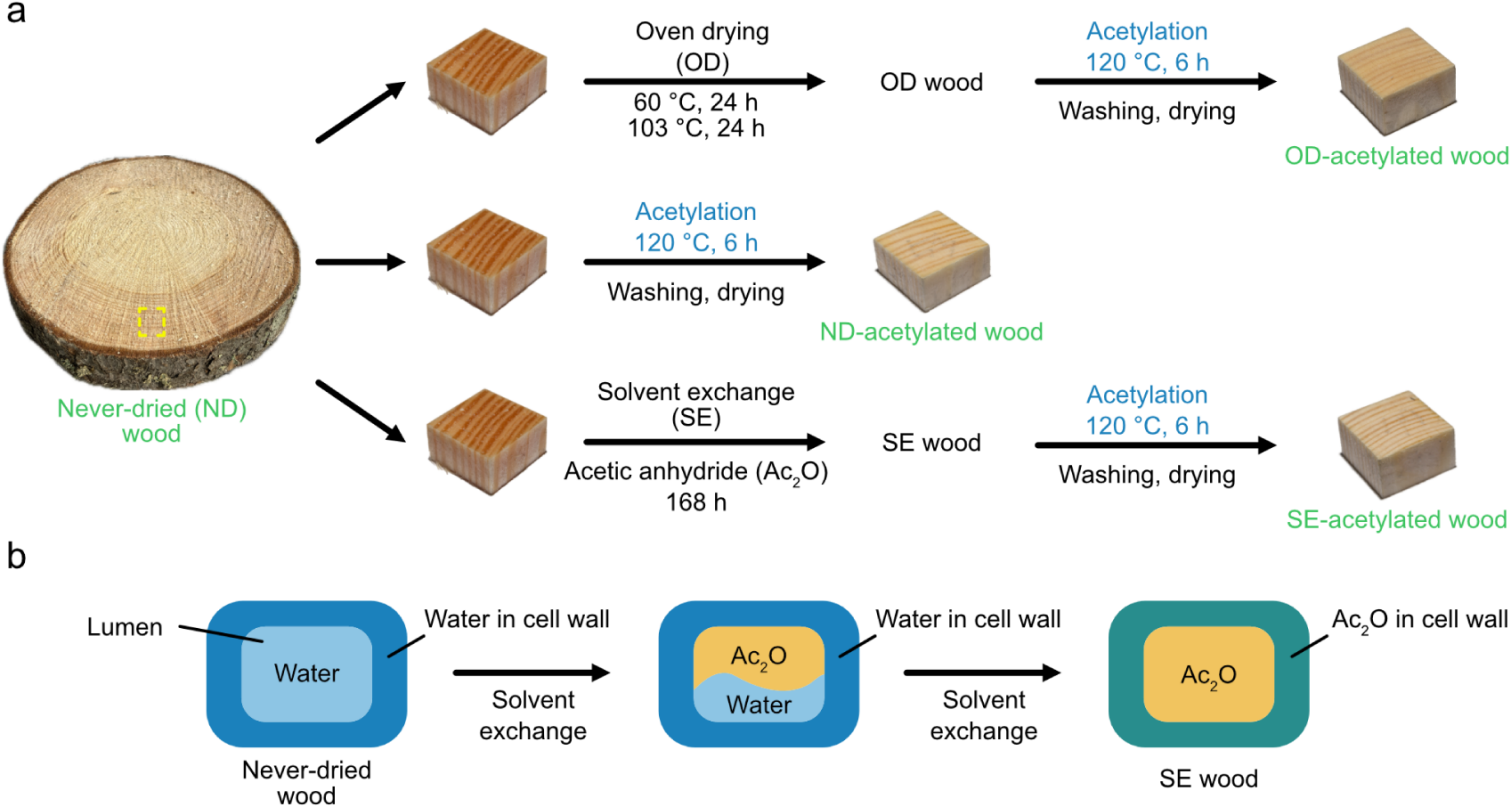
Schematic illustrations of a) the preparation of acetylated wood with various pretreatments, and b) the solvent exchange process for preparing solvent-exchanged wood (SE wood) saturated with Ac_2_O.

## Experimental Section

### Materials

A never-dried Scots pine tree was collected from the Fiskarin Laatupuu Oy sawmill (Fiskari, Finland) in winter and processed on the same day. Acetic anhydride (analytical grade) and sodium acetate (≥99.0%) were purchased as AnalaR NORMAPUR products from Avantor, Inc. (Radnor, PA, USA) under the VWR Chemicals supplier brand. Acetic acid (glacial), hydrogen peroxide (35% w/w), and sodium butyrate (98.5%) were purchased from Merck KGaA (Darmstadt, Germany) under the Sigma-Aldrich brand. All chemicals were used as received, without any further purification.

### Specimen Preparation and Acetylation

Specimens were prepared from a single cross-sectional disc (diameter: ∼34 cm; thickness: 6 cm) cut from never-dried Scots pine sapwood. Each cuboidal wood block, with nominal dimensions of 10 × 20 × 20 mm^3^ (longitudinal × radial × tangential), was cut from the same wood section (replicates) to minimize specimen variability. Six specimens were used for each pretreatment and were also prepared from an adjacent reference group sawed from the wood disc per pretreatment. That is, there were three pretreatment specimen groups and two corresponding adjacent reference groups, each assumed to have comparable biological and physicochemical characteristics to their corresponding pretreatment group. The main specimens were then acetylated with neat Ac_2_O under the following three pretreatment conditions (Figure 1a):

#### Oven-Dried (OD) Specimens

Never-dried wood specimens had their initial masses and dimensions taken and were then sequentially dried at 60 °C for 24 h and at 103 °C for 24 h, followed by storage in a desiccator containing fresh silica gel for 1 h, after which their initial oven-dry masses and dimensions were obtained. Afterward, the specimens were kept in the desiccator just before acetylation and labeled as OD wood. After acetylation, these specimens were referred to as OD-acetylated wood. The adjacent reference group for the OD-acetylated wood was sawn from between it and the ND-acetylated wood, both of which originated from the never-dried wood disc. That is, the OD reference and ND reference involved the same specimens.

#### Never-Dried (ND) Specimens

Never-dried wood specimens were used without prior drying, with their initial never-dried masses and dimensions being recorded. The specimens were labeled as the ND wood. After acetylation, these specimens were termed ND-acetylated wood.

#### Solvent-Exchanged (SE) Specimens

Never-dried specimens, with prerecorded initial masses and dimensions, were immersed in 150 mL of neat Ac_2_O (below the solvent surface) continuously for 7 days at room temperature and ambient pressure, allowing for the gradual replacement of water in the wood by Ac_2_O via diffusion-based SE (Figure 1b), labeled as SE wood. After acetylation, these specimens are termed SE-acetylated wood. The adjacent specimens of the SE-acetylated wood were separated from the OD-/ND-acetylated wood reference specimens.

Acetylation was performed in a round-bottom flask with 150 mL of neat Ac_2_O at 120 °C for 6 h under constant reflux and continuous magnetic stirring at 100 r min^–1^. After acetylation, the specimens in the round-bottom flask were cooled in an ice bath for 15 min, then immersed in acetone for 10 min to stop the reaction. After stopping the reaction, an initial vacuum impregnation with deionized water was performed for 30 min, followed by 1 h of vacuum release and a second 30-min vacuum impregnation using the water-soaking method to initiate the leaching procedure.^20,21^ To remove residual chemicals and soluble components from both unmodified reference and modified main specimens, all specimens were leached with deionized water, with daily water changes, until the rinsing water conductivity decreased to <2 μS cm^–1^ (after 7 days).

The effect of the solvent-exchange process on the potential extraction of cell wall polymers was determined by analyzing the residue liquids for extractives. These were collected after solvent exchange and/or acetylation for further analysis. Solvents in the residues were removed through rotary evaporation using a Büchi Rotavapor R-210 equipped with a Vacuum Controller V-850, Heating Bath B-491, and a Vacuum Pump V-700 (BÜCHI Labortechnik AG, Flawil, Switzerland). The total dry masses obtained were 280 mg (OD) and 560 mg (SE). Samples (60 mg) of the isolated solids from (**1**) OD-acetylated wood and (**2**) SE-acetylated wood were dissolved in CDCl_3_ for nuclear magnetic resonance (NMR) spectroscopic analyses. The measurements were conducted using a Bruker Avance NEO 400 MHz NMR spectrometer (Bruker Corp., Billerica, MA, USA) equipped with a 5 mm BBFO (Broad Band Fluorine Observe) iProbe. The ^1^H spectra were collected with the zg30 pulse sequence with 16 scans and a spectral width (SW) of 16 ppm. The ^1^H–^13^C heteronuclear single-quantum coherence (HSQC) correlation spectra were collected using hsqcetgpsisp.2 pulse sequence, an SW of 11 ppm in F2 (^1^H) dimension with 1024 data points, and 215 ppm in F1 (^13^C) dimension with 128 data points. The spectra were referenced to the solvent residual CHCl_3_ signal at 7.26/77.0 ppm and processed using Bruker TopSpin 4.5.0 software.

### Weight Percentage Gain and Volume Retention

Determining the actual weight percentage gain (WPG) for the acetylated never-dried wood specimens was not feasible because the specimens were used directly for solvent exchange or acetylation without weighing the initial oven-dry mass. To address this issue, the average moisture content (MC) of never-dried wood was first determined by oven drying (60 °C for 24 h and 103 °C for 24 h) a set of adjacent specimens, serving as a reference for each pretreatment group, as previously established. This average MC value was then used to estimate the initial oven-dry mass of the modified wood specimens in the unmodified state via a back-calculation. The individual specimen MC (*u*) [%], from which the averages were determined, was calculated according to eq 1:

1 $$u=\frac{M-M_{0}}{M_{0}}\times 100$$

where *M* is the mass [g] of a never-dried reference wood specimen and *M*_0_ the mass [g] of an oven-dried reference wood specimen. The WPG was calculated using the estimated oven-dry mass of unmodified wood for the never-dried or solvent-exchanged specimens, allowing a reasonable assessment of mass changes resulting from the modification process. Following the deionized water leaching procedure above, the specimens were dried at 60 °C for 24 h, then at 103 °C for an additional 24 h. Afterward, the oven-dried specimens were stored in a desiccator with fresh silica gel for 1 h. These dried specimens were then used to determine both WPG and volume retention (VR). The WPG [%] was calculated using eq 2:

2 $$\mathrm{WPG}=\frac{M_{m}-M_{u}}{M_{u}}\times 100$$

where *M*_m_ is the oven-dry mass [g] of the modified wood, and *M*_u_ is the estimated (never-dried, solvent-exchanged) or actual (conventional oven-drying) oven-dry mass [g] of the unmodified wood.^22^ The individual WPGs were averaged. The VR [%] was calculated as per eq 3:

3 $$\mathrm{VR}=\frac{V_{m}}{V_{u,\mathrm{ND}}}\times 100$$

where *V*_m_ is the oven-dry volume [mm^3^] of the modified wood, and *V*_u,ND_ is the volume [mm^3^] of the unmodified ND wood (never-dried wood). The individual VR values were averaged.

### Anti-Swelling Efficiency

To determine the antiswelling efficiency (ASE) of the acetylated specimens, oven-dried specimens of acetylated and nonacetylated (reference specimens) wood with known dimensions were subjected to vacuum impregnation with deionized water for 30 min, followed by 1 h of vacuum release and a second 30-min vacuum impregnation using the water-soaking method.^20,21^ The water-impregnated wood was further soaked in deionized water to allow it to reach an equilibrium volume.^20,21^ The water was changed daily, and dimensions were measured every 3 days. After 24 days, the wood volume reached equilibrium, with <0.02 mm dimensional changes (absolute value of the mean dimensional change) between successive measurements.^23^ The specimens were then oven-dried sequentially at 60 °C for 24 h and 103 °C for 24 h, followed by storage in a desiccator containing fresh silica gel for 1 h before final dimensional measurements. The volumetric swelling coefficient (*S*) [%], which describes the dimensional stability of the wood, was first calculated (eq 4) so that the ASE parameter was determinable (eq 5).

$$S\;=\frac{V_{\mathrm{ws}}-V_{\mathrm{od}}}{V_{\mathrm{od}}}\times 100$$ 4

where *V*_ws_ is the water-swollen volume [mm^3^] and *V*_od_ the oven-dry volume [mm^3^] of the wood, respectively.^22^ The ASE [%] determines the increase in dimensional stability and was calculated according to eq 5:

5 $$\mathrm{ASE}\;=\;\frac{S_{u}-S_{m}}{S_{u}}\times 100$$

where *S*_u_ is the swelling coefficient [%] of unmodified wood, and *S*_m_ is the swelling coefficient [%] of modified wood.^22^ The individual ASEs were then averaged.

### Determination of Acetyl Content in Wood and Holocellulose by High-Performance Liquid Chromatography

#### Acetyl Content Determination in Wood

A mass of 0.1 g of refined wood powder, prepared using a Fritsch Planetary Mono Mill PULVERISETTE 6 classic line ball mill (Fritsch GmbH, Idar-Oberstein, Germany) with a milling procedure involving 550 r min^–1^ for 30 min, was accurately weighed. The sample was then dried in an oven at 103 °C for 4 h, and the exact dry mass was measured. Subsequently, 0.01 g of sodium butyrate was added to the wood powder as an internal standard. The saponification of the wood powder was carried out in this mixture using 10 mL of 0.2 M NaOH at room temperature under constant stirring for 24 h. After saponification, the mixture was filtered through a cellulose acetate membrane filter (pore size: 5 μm, Merck Millipore, Burlington, MA, USA). A volume of 2 mL of the filtrate was further passed through a polytetrafluoroethylene syringe filter (pore size: 0.22 μm, Phenomenex Inc., Torrance, CA, USA) and transferred to a high-performance liquid chromatography (HPLC) autosampler vial for analysis.

The HPLC analysis was performed using a Thermo Scientific^TM^ Dionex^TM^ UltiMate^TM^ 3000 Standard Systems instrument (Model: WPS-3000, Thermo Fisher Scientific Inc., Waltham, MA, USA) equipped with a data collector and integrator. The results were acquired and processed using Chromeleon^TM^7 Chromatography Data System software [Version 7.2.10 (23925)]. Separation of AcOH was achieved on a Phenomenex Rezex Organic Acid H^+^ ion exchange column (300 × 7.8 mm). The mobile phase consisted of 0.0025 M H_2_SO_4_ with a flow rate of 0.5 mL min^–1^, and the sample injection volume was 10 μL. Detection was carried out at 210 nm. An external standard calibration curve was constructed using four concentrations of sodium acetate (400–2400 ppm), with sodium butyrate as the internal standard. All standard solutions were prepared in the same 0.2 M NaOH solution used for saponification of the wood powder. The individual results were averaged.

#### Acetyl Content Determination in Holocellulose

Holocellulose was extracted from sliced wood specimens using a 1:1 (v/v) mixture of AcOH and hydrogen peroxide at 80 °C for 6 h to remove the lignin. Complete lignin removal from the holocellulose was confirmed by attenuated total reflectance (ATR) Fourier-transform infrared (FTIR) spectroscopy (Figure S1, Supporting Information). The acetyl content of the holocellulose was determined by HPLC, using the same sample preparation, saponification, filtration, and analytical conditions as those used for the wood powder. The individual results were averaged.

### Attenuated Total Reflectance Fourier-Transform Infrared Spectroscopy

Wood pellets, prepared from wood powder milled using a Fritsch PULVERISETTE 6 ball mill, were utilized for ATR-FTIR spectroscopic analysis. Spectra were acquired using a spectrometer with a Quest ATR accessory (Specac Ltd., Orpington, England), equipped with a diamond crystal (Spectrum Two FT-IR Spectrometer; PerkinElmer, Inc.; Waltham, MA, USA), covering the wavenumber range of 4000–400 cm^–1^, at a resolution of 4 cm^–1^ with 16 accumulations. The spectra were baseline-corrected using the anchor-point approach with the second derivative (zeroes) method, adjacent-averaging smoothing used, smoothing window size: three, threshold: 0.05, connection by line interpolation with spectrum snapping active, and then normalized at approximately 1030 cm^–1^, corresponding to C–O valence vibrations originating from cellulose, hemicelluloses, and lignin.^24,25^

### Confocal Raman Microspectroscopy

Confocal Raman microspectroscopy was performed on both intact and delignified wood specimens. For intact specimens, transverse sections were cut from the center of the wood blocks using a manual rotary microtome (Nahita ZFP011, Auxilab S.L., Beriáin, Spain). The sections, with thicknesses of 20–30 μm, were placed on glass slides in a droplet of deionized water, then covered with a glass coverslip (0.17 mm thick) and sealed with nail polish. Raman imaging was performed using a confocal Raman microscope (Renishaw^Ⓡ^ inVia^TM^ QONTOR^Ⓡ^, Renishaw plc, Wotton-under-Edge, England) equipped with a 532 nm diode laser and a Centrus 05TJ55 charge-coupled device detector, positioned behind an 830 lines mm^–1^ grating. Measurements were conducted over a spectral range of 97–3667 cm^–1^ at 50 mW laser power, using a 63× water-immersion objective lens [numerical aperture (N.A.): 1.20], with an acquisition time of 0.2 s and a single accumulation per spectrum. Raman images contained 220 × 220 points per line, with a step size of 0.2 μm.

For the delignified specimens, transverse surfaces of nonacetylated and acetylated wood blocks (20 × 20 × 10 mm^3^) were first smoothed using a sliding microtome (Swiss Federal Institute for Forest, Snow and Landscape Research WSL Lab-Microtome, Sandro Luchinetti, Schenkung Dapples, Zürich, Switzerland). The smoothed surfaces were then delignified using a 1:1 (v/v) mixture of AcOH and hydrogen peroxide at 80 °C for 4 h, as previously reported.^26,27^ Residual chemicals were removed by washing the specimens with deionized water until the rinsing water conductivity decreased to <2 μS cm^–1^. Wood blocks were placed in a Petri dish filled with deionized water, with the smoothed transverse surface just above the water level. A few drops of deionized water were applied to the smoothed surface, which was then covered with a quartz microscope slide. Raman imaging was performed using the same microscope, this time equipped with a 785 nm laser source, the same charge-coupled device detector, and an 830 lines mm^–1^ grating. Measurements were conducted over the spectral range of 700–2260 cm^–1^ using a 50× objective lens (N.A.: 0.50), with an acquisition time of 0.8 s and one accumulation per spectrum. The laser power was 100 mW. The Raman images contained 150 × 150 points per line, with a step size of 0.3 μm. This block-versus-section strategy follows our previously validated protocol, where a 785 nm near-infrared diode laser was also used.^28^

### Image Processing and Principal Component Analysis

Cosmic rays were selectively removed using automatic detection via the nearest neighbor (noise level scaling factor: 10.0, spectrum height scaling factor: 50%) and width of features (width parameter: 3, height parameter: 15.0) algorithms in WiRE^TM^ 5.7 (build 44973) software (Renishaw plc). Wavenumbers outside the range of 300–3600 cm^–1^ were excluded before spectral processing. Average spectra were obtained from the cell wall and cell corner regions of latewood tracheids. For each sample image, four regions of interest (ROIs) were manually selected at predefined locations, and the mean spectrum of each ROI was calculated. The spectral data were subjected to polynomial baseline correction (degree, *n* = 6). Area-integrated images were generated for both intact and delignified specimens. For intact specimens, the spectral regions of 1552–1699 cm^–1^ and 1714–1772 cm^–1^ were selected, while for delignified specimens only the 1701–1771 cm^–1^ region was used. In both cases, a polynomial baseline correction (*n* = 1) was applied before numerical integration using the trapezoidal method, which approximates the area under the curve by dividing it into trapezoids.^29^ The resulting area values were then folded back to the original image dimensions. Pixel-intensity histograms were also generated from the area-integrated images for each intact sample.

For multivariate analysis, the three-dimensional image hypercubes from never-dried (nonacetylated) wood, SE-acetylated wood, and OD-acetylated wood were fused into a single image mosaic. The spectral range was limited to 300–3600 cm^–1^, and the data were transformed into two-dimensional arrays, with rows corresponding to individual pixel spectra and columns to wavenumber values. The spectra were preprocessed using polynomial baseline correction (*n* = 6), followed by vector normalization and mean centering. Principal component analysis (PCA) was performed using singular value decomposition.^30^ The PCA was first applied to identify and remove lumen water pixels using a score threshold. The remaining pixel indices were then used to extract the corresponding normalized spectra, which were mean-centered using the updated mean. The PCA was reapplied to the selected pixels. The resulting score values were folded back to the original image dimensions and interpreted alongside their respective loading vectors. All data processing was performed using MATLAB R2025a (The MathWorks, Inc.; Natick, MA, USA). Spectral preprocessing was performed using commercially available functions from the PLS Toolbox (Eigenvector Research, Inc.; Manson, WA, USA).

## Results and Discussion

### Weight Percentage Gain, Volume Retention, and Anti-Swelling Efficiency

The WPG values indicated the differences in the degree of modification among the specimens with different pretreatment conditions. Both the SE-acetylated wood and the OD-acetylated wood exhibited higher WPGs (19.5% and 19.7%, respectively) than the ND-acetylated wood (16.7%). The difference in the WPG of ND-acetylated wood may be due to a high MC (109.6 ± 7.7%) of natural origin, with the free water in the wood resisting the diffusion of Ac_2_O into the wood during the relatively short contact time of 6 h. The comparable WPGs of SE- (99.6 ± 5.5% MC) and OD-acetylated wood (111.8 ± 5.4% initial MC) indicate that both approaches provided favorable acetylation conditions. Acetic anhydride pretreatment facilitated the diffusion of the reagent into the cell walls of SE wood, thereby improving the acetylation degree.

Volume retention revealed the volumetric shrinkage of the modified wood specimens relative to their never-dried state. A higher VR value indicates greater permanent cell wall bulking in wood due to acetylation, as was also observed under optical microscopy (Figure S2, Supporting Information). After acetylation, all acetylated specimens showed a reduced volume relative to their original, never-dried state. Although the SE- and OD-acetylated wood had nearly identical WPGs, the SE-acetylated wood exhibited a higher VR (96.0% compared to 93.8%). Drying before acetylation partially collapsed the cell wall porosity and restricted the diffusion of Ac_2_O into the cell wall. As a result, acetylation in dried wood likely occurred mainly near the lumen surfaces, where diffusion distances were shortest, with additional reactions occurring in deeper cell wall regions, where drying-induced microcracks offered alternative pathways, leading to a less uniform modification.^31^ In contrast, the solvent-exchange process allowed Ac_2_O to replace water in never-dried wood more gradually and uniformly, thereby improving reagent accessibility and yielding greater permanent cell wall bulking, as reflected in the higher VR. As excess moisture in the lumina and other pores hindered Ac_2_O penetration in never-dried specimens and decelerated reaction kinetics, the ND-acetylated specimen exhibited the lowest VR (92.3%). The higher WPG and VR values in SE-acetylated wood directly correspond to an improved ASE.^32,33^ The ASE (Table 1) of the acetylated specimens depends on both the overall degree of acetylation and its spatial distribution. The SE-acetylated wood exhibited the highest ASE, followed by OD- and ND-acetylated wood, consistent with their VR results. In addition, the degree of acetylation and the acetylation ratio between holocellulose and lignin may also contribute to these effects and have been investigated by HPLC and NMR analyses.

**Table 1:** Physical Properties of Non-Acetylated and Acetylated Wood with Different Pretreatments^a^

Specimen	WPG (%)	VR (%)	ASE (%)	Density (kg m^–3^)^b^
Nonacetylated	–	85.8 (0.9)	–	566 (27)
Never-dried (ND)	16.7 (2.7)	92.3 (0.9)	53.2 (4.3)	612 (25)
Solvent-exchanged (SE)	19.5 (3.6)	96.0 (1.0)	72.8 (3.5)	623 (12)
Oven-dried (OD)	19.7 (0.6)	93.8 (1.0)	57.8 (6.1)	613 (22)

a Values in parentheses are sample standard deviations, *n* = 6 (WPG, VR, and density) or *n* = 5 (ASE).

b Oven-dried conditions.

### Acetyl Content in the Specimens

To determine the acetyl content in the specimens, acetate ions were released by saponification and quantified by HPLC.^34^ As shown in Table 2, the ND-acetylated wood (3.92 mmol g^–1^) contained much less acetyl content compared to the SE- (4.54 mmol g^–1^) and OD-acetylated (4.63 mmol g^–1^) wood, a result consistent with the WPG values. Additionally, to distinguish the acetyl content in lignin and holocellulose, lignin was removed from the wood, and the resulting acetylated holocellulose was collected and subsequently saponified. The HPLC analysis of the holocellulose revealed that the OD-acetylated wood (3.57 mmol g^–1^) contained more acetylated holocellulose than the SE-acetylated wood (2.44 mmol g^–1^), suggesting that acetylation in the OD wood occurred preferentially in the holocellulose. In contrast, a higher degree of lignin acetylation was observed in SE-acetylated wood. Shi et al.^35^ recently reported a preferential association of ethanol with lignin during competitive sorption from a water/ethanol mixture, suggesting that the solvent polarity might affect the spatial pattern of wood modification. The greater extent of lignin modification observed here in SE-acetylated wood provides experimental evidence in support of this hypothesis.

**Table 2:** Acetyl Group Content as Characterized by Saponification and HPLC^a^

	Acetyl group content (mmol g^–1^)	
Sample	Wood	Holocellulose
Nonacetylated	0.44 (0.03)	0.74 (0.01)
ND-acetylated	3.92 (0.04)	1.85 (0.02)
SE-acetylated	4.54 (0.06)	2.44 (0.02)
OD-acetylated	4.63 (0.07)	3.57 (0.01)

a Values in parentheses are sample standard deviations, with *n* = 3 (wood) or *n* = 2 (holocellulose).

### Nuclear Magnetic Resonance Analysis of the Extractives

In all acetylation experiments, low-molecular-weight extractives dissolved into Ac_2_O, as indicated by the observed color change of the submersion (solvent exchange) or reaction medium. After each experiment, the residual solvent-exchange solvent component soluble fraction, or reagent with the soluble fraction, was recovered and removed. Further analysis of the recovered extracts from OD-acetylated wood and SE-acetylated wood residues by NMR spectroscopy showed that they consist predominantly of fatty acids and terpenoids (Figure 2), which are typical softwood (Scots pine) extractives (cf. Figure S3 for reference HSQC correlation spectra). Evidence for low-molecular-weight lignin fragments was observed in the OD residue, including aromatic guaiacyl signals and resonances attributable to β-*O*-4 side-chain structures. No signals attributable to carbohydrate-derived species were detected in the analyzed residues, indicating that the acetylation did not solubilize holocellulose fragments.

**Figure 2:**
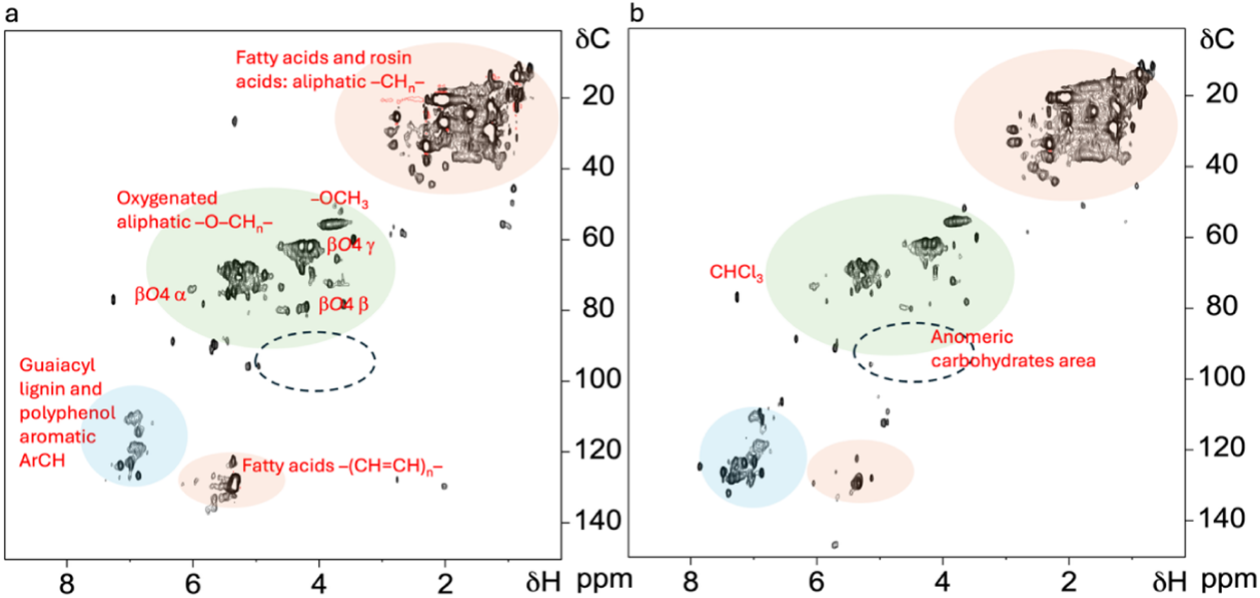
Nuclear magnetic resonance spectroscopic results of the ^1^H–^13^C HSQC correlation spectra type for a) OD residues, and b) SE residues. The detected chemical components or functionalities have been pointed out. The dashed circle shows the absence of carbohydrate signals.

### Attenuated Total Reflectance Fourier-Transform Infrared Spectroscopy

Furthermore, ATR-FTIR spectroscopy was used to analyze the chemical structure of acetylated wood. The acetylated wood did not show any new peaks in comparison to the nonacetylated (dried, unmodified) wood (Figure 3a). However, numerous peak intensities of the acetylated wood were enhanced in contrast to the unmodified wood due to the introduction of ester groups [−O–(C=O)–CH_3_] via the conversion of the −OH groups to these ester functionalities. Significant enhancements were observed at wavenumbers of 1735, 1370, and 1220 cm^–1^. The increased intensity of the nonconjugated ester carbonyl C=O stretching band at 1735 cm^–1^ in acetylated specimens,^24^ compared with the nonacetylated wood, indicated that acetylation had occurred at the −OH groups in wood, as evidenced also by the decreased −O–H stretching intensity (∼3400 cm^–1^).^36^ The OD-acetylated and SE-acetylated wood showed similar degrees of acetylation, both of which exceeded that of the ND-acetylated wood. In nonacetylated wood, ∼1370 cm^–1^ is attributed primarily to C–H bending (deformation) in the glucose units of the cellulose or hemicelluloses^37,38^ and symmetric deformation vibration of C–H in the lignin methoxyl (−OCH_3_) group.^39^ After acetylation, the intensity at the C–H band increased due to overlap with the C–H vibration of the acetyl group. The 1225 cm^–1^ band in wood arises from both C–O and C=O stretching vibrations related to the guaiacyl aromatic rings of lignin.^25,40,41^ For the C=O, these vibrations occur in the 1-substituent position (α carbon) of the guaiacyl aromatic rings.^45^ The increased intensity of the 1225 cm^–1^ band after acetylation is due to the increase in C–O and C=O bond concentration caused by the introduction of acetyl groups in lignin and holocellulose. Overall, the ATR-FTIR results indicate that the extents of acetylation in the OD- and SE-acetylated wood were similar and higher than in the ND-acetylated wood.

**Figure 3:**
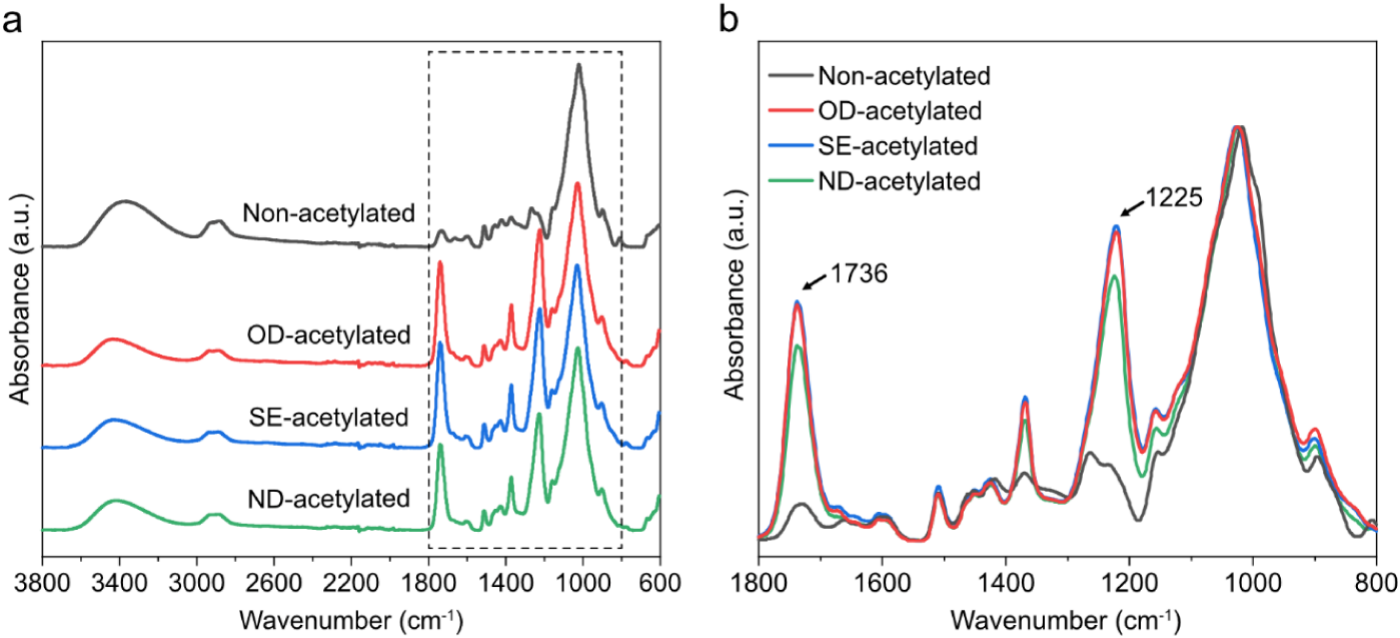
Fourier-transform infrared spectra of nonacetylated and acetylated wood in the range of a) 3800 to 600 cm^–1^ and b) 1800 to 800 cm^–1^.

### Confocal Raman Microspectroscopy

#### Cell Walls and Cell Corners Mean Raman Spectra

The mean Raman spectra of the cell wall (CW) and cell corner (CC) regions are presented in Figure 4a and 4b, respectively. The nonacetylated reference spectra exhibit the characteristic Raman features of wood in both regions (band assignments are summarized in Tables S1 and S2). In band 1 (1500–1800 cm^–1^), acetylation-related spectral changes are evident in both CW and CC regions. The peak at 1604 cm^–1^, assigned to the aromatic C=C stretching vibration of lignin, appears to be affected by acetylation in both regions. A notable change is observed at ca. 1663 cm^–1^, particularly in the SE- and OD-acetylated specimens. Furthermore, a new band emerges at ca. 1735 cm^–1^ (ND, SE, and OD) in the CW spectra, assigned to the ester carbonyl stretching, ν­(C=O), of the introduced acetyl groups.^42^ In the CC spectra, the corresponding band appears at 1735 cm^–1^ (ND, SE) or 1739 cm^–1^ (OD), suggesting a slightly different chemical environment for the carbonyl groups in the lignin-rich middle lamella. In band 2 (2800–3100 cm^–1^), a peak at ca. 2942 cm^–1^ increases in intensity with the increasing degree of acetylation in both regions. This band is assigned to the CH_3_ asymmetric stretching of the introduced acetyl groups.^43–47^ In the CC spectra, the shoulder near 2892–2899 cm^–1^ in the nonacetylated state, assigned to C–H stretching vibrations of CH_3_ and OCH_3_ groups in lignin, shifted to a distinct peak at 2942 cm^–1^ upon acetylation, reflecting newly introduced acetyl CH_3_ contributions.^43–47^

**Figure 4:**
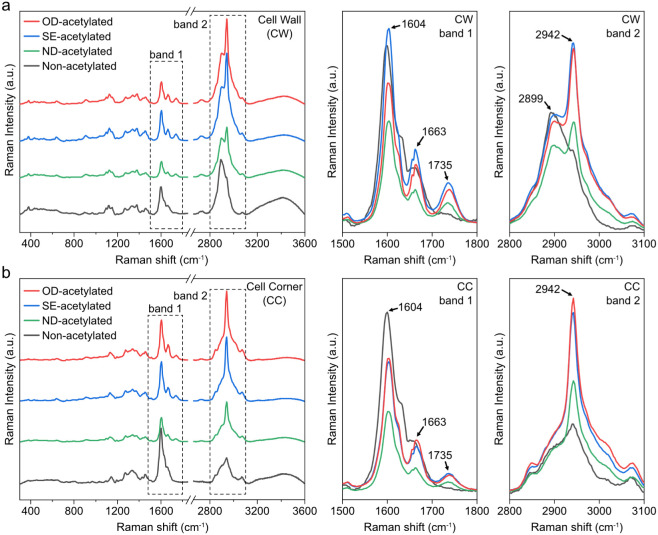
Mean Raman spectra of nonacetylated wood and acetylated wood collected from a) cell wall (CW) regions, and b) cell corner (CC) regions.

#### Band Integration of Lignin and Acetylation-Related Chemical Features

Selective spectral regions associated with lignin and acetylation-related chemical features were integrated over the defined wavenumber ranges and transformed into integrated intensity images (Figure 5a). The integrated regions comprise (A) 1552–1699 cm^–1^, corresponding to the aromatic C=C stretching vibrations characteristic of lignin, and (B) 1714–1772 cm^–1^, corresponding to the ester carbonyl (C=O) region indicative of acetylation.^28^ In the nonacetylated reference, saturated pixels in certain cell corner regions are attributed to spectral artifacts. The SE-acetylated cell appears to retain a swollen morphology, likely because of Ac_2_O persisting after the elevated reaction temperature and subsequent oven drying. In contrast, the ND- and OD-acetylated sections exhibit less swollen middle lamella regions, suggesting that cellular shrinkage occurred during the reaction and oven drying. Furthermore, the intersample comparison in (B, 1714–1772 cm^–1^) reveals clear differences in acetylation. The ND-acetylated section shows similarly low integrated intensity values, which may reflect spatial variability in the distribution of acetyl groups within the specimen. However, the bulk WPG (Table 1) and acetyl group content (Table 2) suggest a somewhat higher overall acetylation degree. The SE-acetylated section shows the highest and most uniform distribution of integrated ester carbonyl intensity values, consistent with a more homogeneous acetylation degree. The OD-acetylated section also shows relatively high integrated intensities, though with a less uniform spatial distribution than the SE-acetylated section. The corresponding pixel intensity histograms confirm these observations in Figure 5b: the SE-acetylated section shows the highest and narrowest distribution, the OD-acetylated section displays a broad but relatively high-intensity distribution, and the ND-acetylated section largely overlaps with the nonacetylated reference.

**Figure 5:**
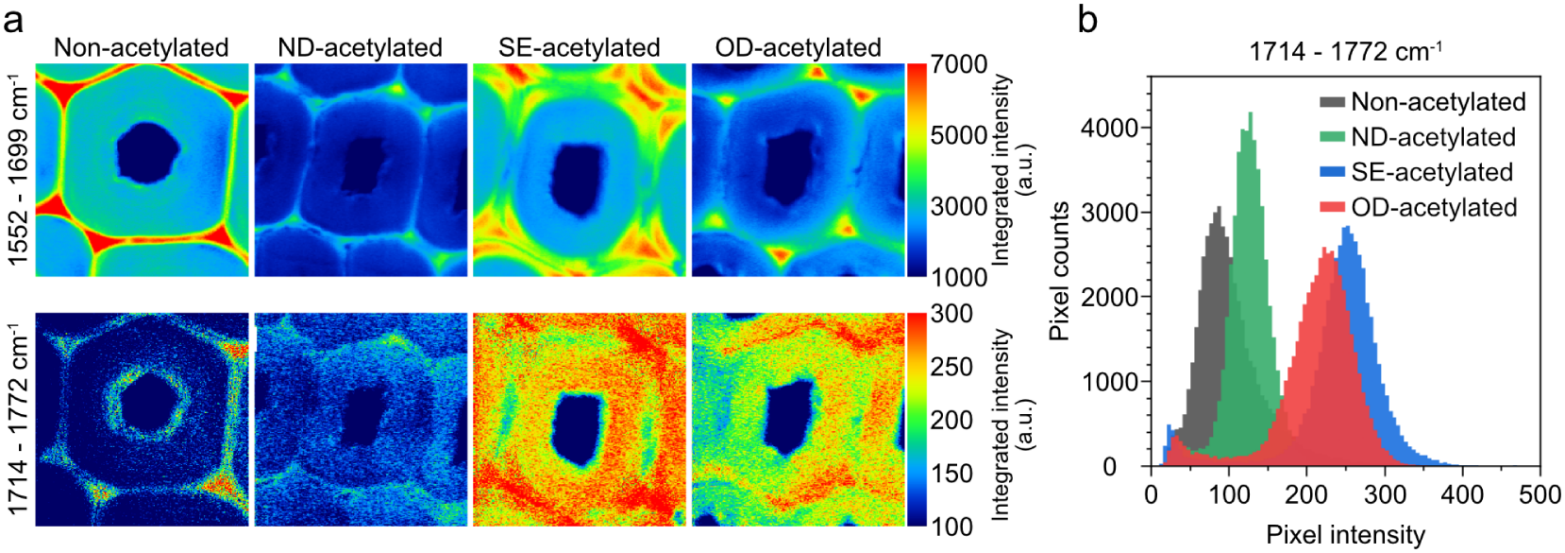
Raman imaging on the cross-sections of nonacetylated (never-dried) and acetylated wood with different pretreatments. a) Integrated intensity value images generated over the spectral regions of 1552–1699 cm^–1^ and 1714–1772 cm^–1^. Each map is the baseline-corrected integrated band area over the stated wavenumber range obtained by trapezoidal integration. b) Pixel intensity histograms of the integrated intensity values from the 1714–1772 cm^–1^ region. Note: The acetyl band histogram is shown as it reflects the treatment. The lignin band only maps lignin distribution, which is already conveyed by the image and is not relevant to the acetylation behavior.

### Principal Component Analysis

The between-section differences in the acetylation degree among the SE- and OD-acetylated wood were analyzed using PCA (Figure 6). Only non-, SE-, and OD-acetylated samples were included in the analysis, as the aim was to highlight differences in acetylation distribution between these untreated and pretreated sections. The first four principal components (PCs) account for 93.0% of the total variation. The PC1 (Figure 6a) explained 64.2% of the variation, with prominent (higher peak contributions) bands at ca. 1589, 1633, 2889, and 2942 cm^–1^. The bands at ca. 1589 ^48^ and 1633 cm^–1^ ^47,49^ belong to lignin, while those at ca. 2889 ^44,50^ and 2942 cm^–1^ ^42^ are related to cellulose/hemicellulose (xylan) and acetylation, respectively. The PC1 score image primarily distinguishes treatment-related changes, with positive bands corresponding to acetylation and negative bands corresponding to native wood components. Specifically, the ca. 2942 cm^–1^ band corresponds to the CH_3_ stretching vibration, which is strengthened by acetylation due to the presence of the CH_3_ functionality in the acetyl groups.^51,52^ Additionally, the band region of ca. 3200–3600 cm−1 ^42^ corresponds to water-related information indicated by leftover lumen pixels. The PC2 (Figure 6b) explained 19.5% of the variation, with loadings highlighting a positive lignin-dominant band (ca. 1600 cm^–1^ ^43,47^ ) and negative cellulose-related spectral features (ca. 1380,^50,53^ 2895 cm^–1^ ^43,45^ ). The PC2 score image separates lignin-rich regions in the cell corners, while cellulose and other chemical constituents are localized in the cell walls. The PC3 loadings exhibit baseline artifacts or extreme pixels (outliers), which are highlighted in the score image. It explains 5.9% of the total variation, mostly separating wood constituents or those of modified wood at the ca. 1608,^43–45,47^ 1666,^43–45,47^ and 2942 cm^–1^ ^42–45,47^ bands, with the ca. 3130–3600 cm^–1^ ^42^ range corresponding to water, from baseline artifacts (see Figure S4). The PC4 (3.2%) also shows artifacts and missing wood-related information, such as the ca. 1604,^43,44,45,47^ 2892,^43,44^ and 2949 cm^–1^ ^42^ bands (Figure S4).

**Figure 6:**
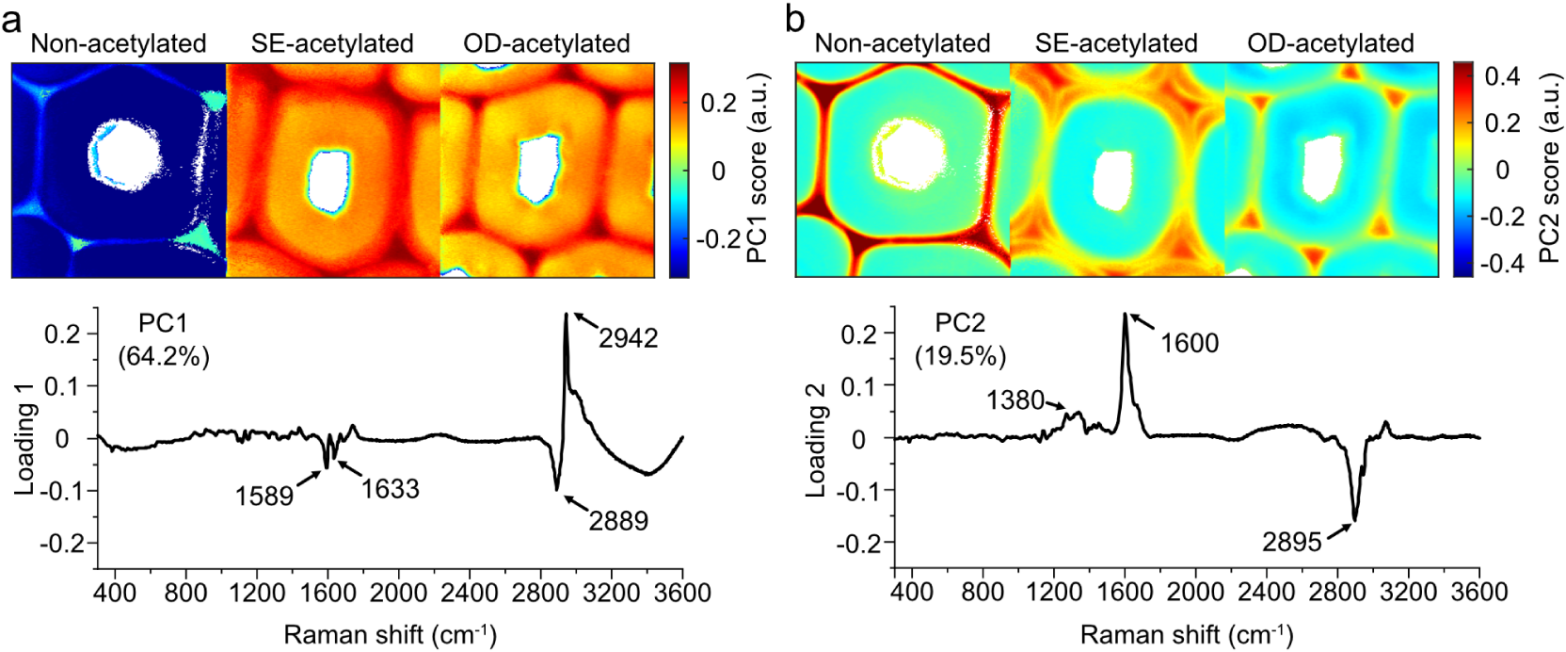
Principal component analysis (PCA) of the Raman imaging data. Scores and corresponding loading vectors of a) PC1, and b) PC2. The PCA scores are dimensionless projections onto the PCs.

Although the SE- and OD-acetylated specimens appeared structurally similar in the PC1 score maps, the SE-acetylated specimen showed stronger and more uniform acetylation within the cell wall, as well as in the middle lamella and cell corners, compared to the OD-acetylated specimen. Particularly strong acetylation was observed near the cell wall of the middle lamella in the SE-acetylated specimen. The higher degree of acetylation adjacent to the middle lamella in the SE-acetylated wood can likely be explained by the higher permeability of swollen, never-dried wood, which facilitates reagent penetration. In PC2, lignin-associated information was particularly intensified near the cell corner of SE-acetylated wood, which can be a result of increased lignin acetylation through solvent exchange. The preferential acetylation of the middle lamella and cell corners restricted swelling in these regions, as well as the outward swelling of the cell wall upon exposure to water, thereby explaining the enhanced ASE observed in SE-acetylated wood.^54^

### Confocal Raman Microspectroscopy of Delignified Wood

The wood specimens were further delignified to elucidate the spatial distribution of acetylation within the holocellulose fraction of the wood cell wall via Raman imaging. The mean Raman spectra extracted from the cell wall region (Figure 7a) confirmed that the holocellulose was acetylated in the acetylated specimens, in agreement with HPLC results. The intensified carbonyl band at 1730 cm^–1^, compared to the nonacetylated reference, evidenced this. The Raman images corresponding to the acetylation band (Figure 7b) indicate that the strongest holocellulose acetylation took place in the OD-acetylated wood. However, the distribution of acetyl groups in OD-acetylated wood was heterogeneous, with a few regions (red spots in Figure 7b) showing exceptionally high intensity compared to the rest of the cell wall. The heterogeneity in OD-acetylated wood could reflect microcracks developed during drying,^31^ in which Ac_2_O penetrated more readily, leading to locally higher acetylation and a stronger overall Raman signal. On the contrary, acetyl groups were revealed to be uniformly distributed in the cell wall of ND- and SE-acetylated wood, although less intensified than in OD-acetylated wood. Hence, the diffusion-driven solvent exchange method represents a novel approach for achieving uniform acetylation of wood cell walls. It could be coupled with self-flow-assisted wood modification^55^ to achieve more efficient and effective wood acetylation, offering a potential route toward a scalable process.

**Figure 7:**
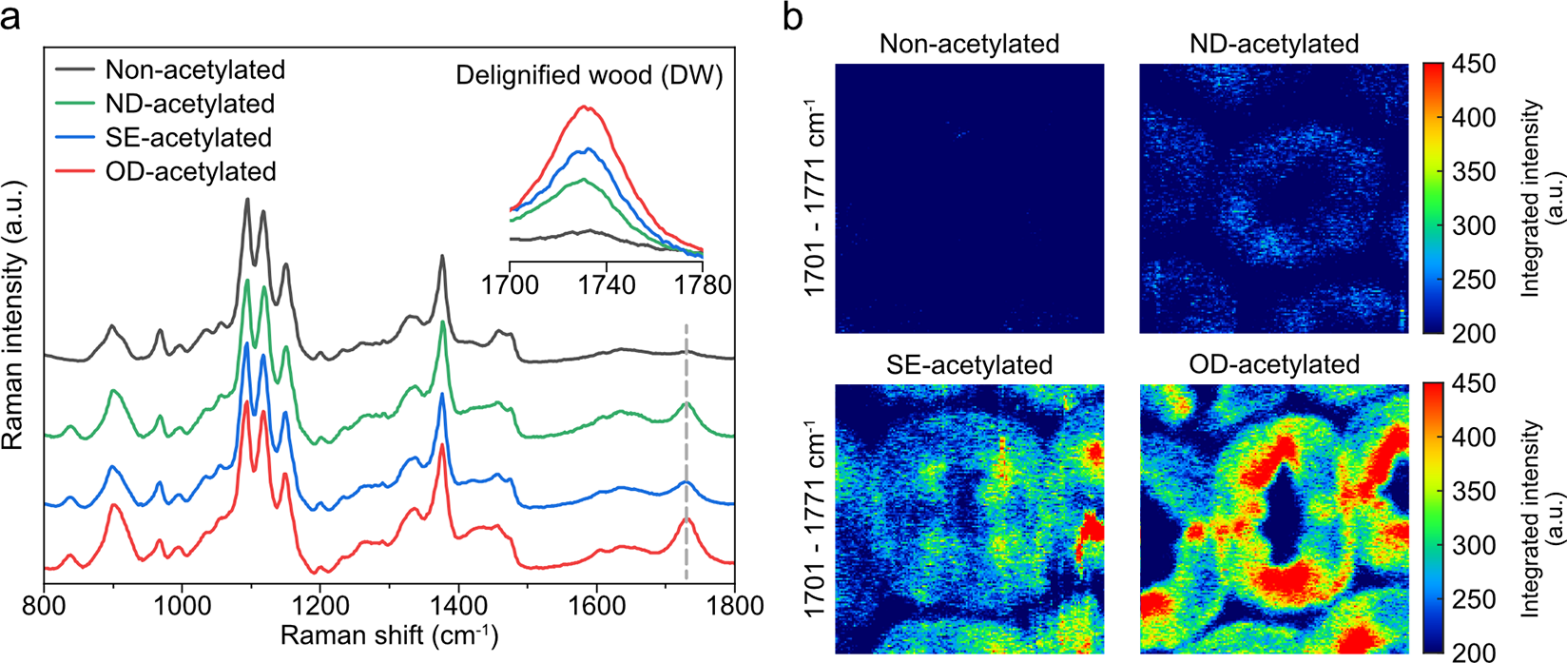
Raman microspectroscopy analysis of nonacetylated and acetylated wood after delignification. a) Average Raman spectra collected from the delignified wood cell wall. b) Raman color map generated by integrating the band of 1701–1771 cm^–1^. Each map is the baseline-corrected integrated band area over the stated wavenumber range obtained by trapezoidal integration.

## Conclusions

Acetylation of never-dried wood after solvent exchange results in a more uniform modification throughout the cell wall and middle lamella of the wood. While the WPG is similar for both SE- and OD-acetylated wood, SE-acetylated wood shows much higher ASE, resulting from increased acetylation in lignin-rich regions such as the middle lamella and cell corners. Raman band-integrated images and PCA confirm a more uniform distribution of acetyl groups and increased lignin accessibility in SE-acetylated wood. The HPLC results show that Ac_2_O diffuses into never-dried wood, which undergoes more comprehensive lignin acetylation, while OD-acetylated wood has greater acetylation in holocellulose. Overall, Ac_2_O prediffusion influences the open structure of never-dried wood, enabling deeper, more consistent acetylation and making it a more thorough wood treatment route.

## Supplementary Material



## Data Availability

The research data can be obtained from Zenodo at doi.org/10.5281/zenodo.20610419. The corresponding NMR dataset is available from the authors upon reasonable request.

## Notes

The ATR-FTIR spectra of the delignified specimens (Figure S1); optical microscopy images of wood samples in the air-dry state (Figure S2); the HSQC NMR correlation spectra of commercial reference samples from crude softwood tall-oil fatty acid (Figure S3); the PC3 and PC4 (Figure S4); band assignments for the mean cell-wall (Table S1) and cell-corner (Table S2) Raman spectra of nonacetylated and acetylated sample sections (PDF)
