# Supplementary material for: Acetylation of Never-Dried Wood via Diffusion-Driven Solvent Exchange
Source: Biomacromolecules. 2026 Jun 11;27(7):4802–12. doi: 10.1021/acs.biomac.6c00768 (PMC13370771; doi:10.1021/acs.biomac.6c00768)
Supplement: Supplementary file 1 [file bm6c00768_si_001.pdf]

Supporting Information

Acetylation of Never-Dried Wood via Diffusion-Driven Solvent Exchange

Md Tipu Sultan[1§], Mikko Valkonen[1§], Muhammad Awais[1*], Paula Nousiainen[1], Kristiina Lillqvist[1], Shennan Wang[1*], Lauri Rautkari[1]

[1]Department of Bioproducts and Biosystems, School of Chemical Engineering, Aalto University, Espoo FI-02150, Finland

[§]M.S. and M.V. contributed equally to this work.

[*]Corresponding authors.

This supporting information contains:

Figure S1 The ATR-FTIR spectra of the delignified specimens.

Figure S2 Optical microscopy images of wood samples in the air-dry state.

Figure S3 The HSQC NMR correlation spectra of commercial reference samples from crude softwood tall-oil fatty acid.

Figure S4 The PC3 and PC4.

Table S1 Band assignments for the mean cell-wall Raman spectra of non-acetylated and acetylated sample sections.

Table S2 Band assignments for the mean cell-corner Raman spectra of non-acetylated and acetylated sample sections.

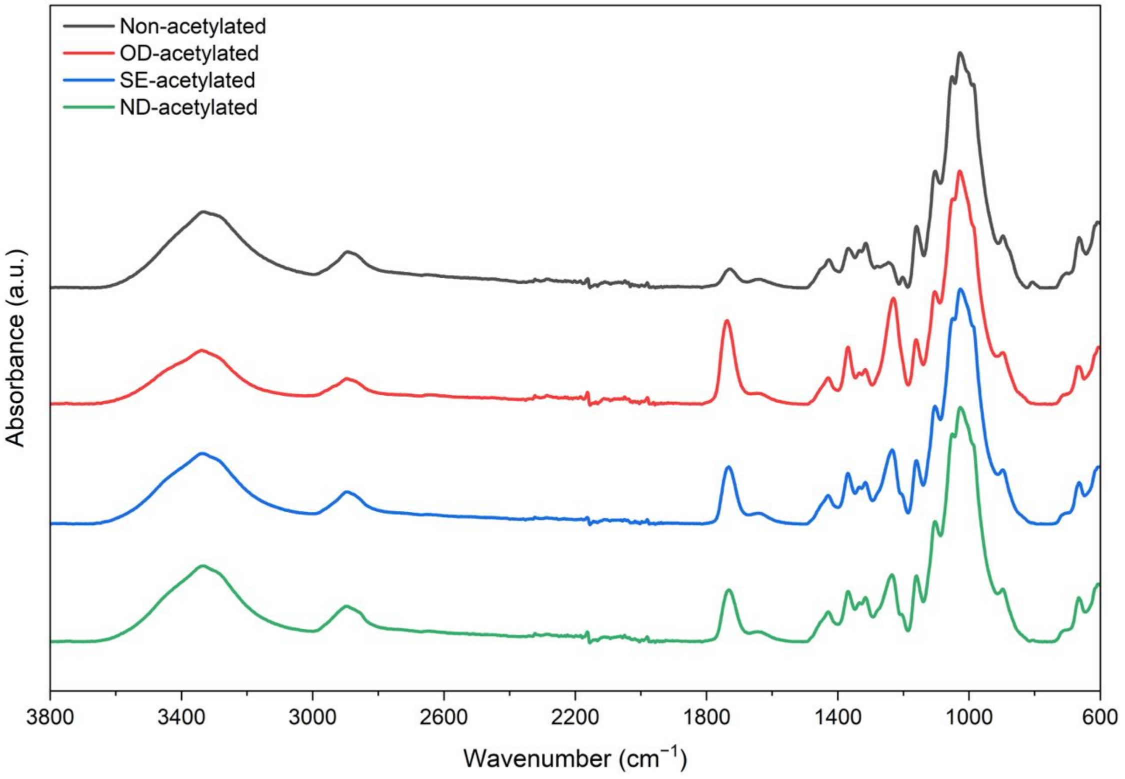

Figure S1. The ATR-FTIR spectra of the delignified specimens. The delignification was observed to have been completed by the significant diminishing or disappearance of the 1662, 1598/1600/1606, and 1509 cm^{-1} lignin-related band peak maxima.[1]

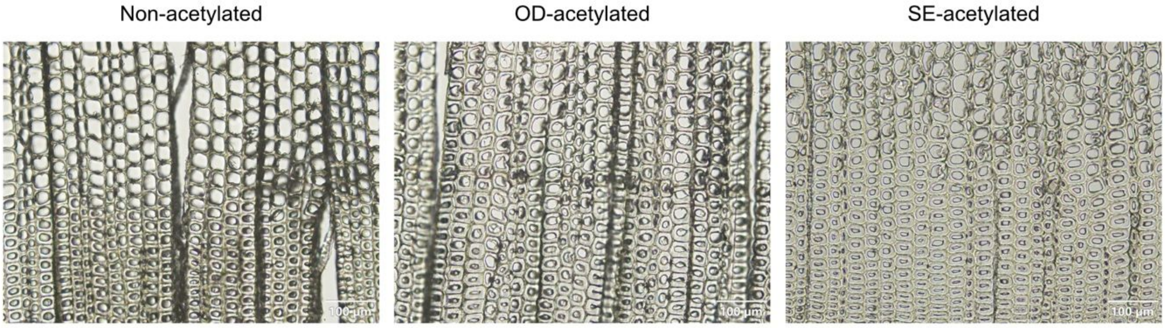

Figure S2. Optical microscopy images of wood samples in the air-dry state.

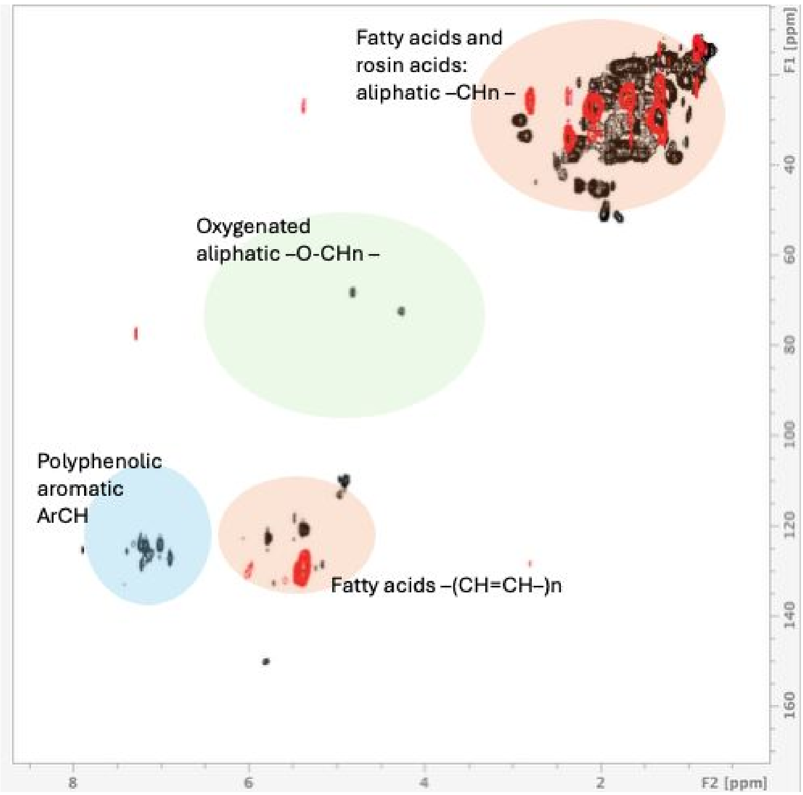

Figure S3. The HSQC NMR correlation spectra of commercial reference samples from crude softwood tall-oil fatty acid from kraft pulping, further refined to distilled fractions by Forchem, Respol Group, Rauma, Finland. Red signals FOR2, with high tall-oil fatty acid content and low rosin acids and unsaponifiables content. Black signals FOR90, an unmodified tall-oil rosin product with a high content of abietic-type rosin acids.

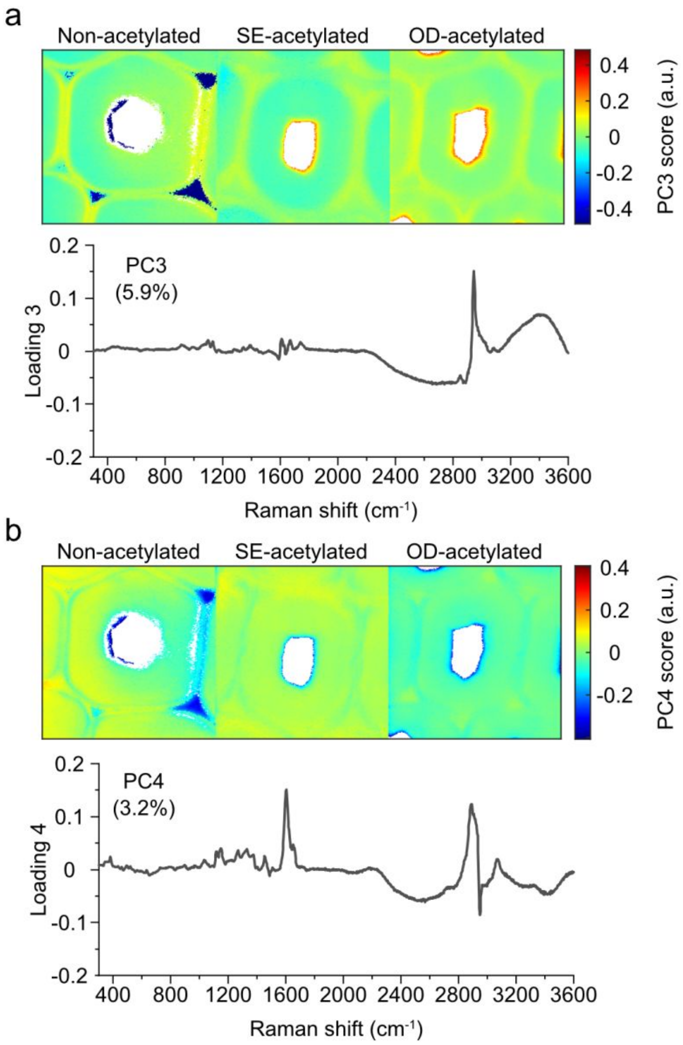

Figure S4. The PC3 and PC4. The PCA scores are dimensionless projections onto the principal components.

Table S1. Band assignments for the mean cell-wall Raman spectra of non-acetylated and acetylated sample sections.

Sample [a]	Component [b]	Raman shift (cm^{-1})	Strength [c]	Functionality	Type [d]	Change (cm^{-1})	Ref.
Non-ac	C	381	m	Some heavy atom, CCC ring	Stretching; CCC: in-plane scissoring	–	2,3
	L	632	w	Skeletons of aromatic rings, substituent groups and side chains	Deformation		2,4
	C	899	vw	HCC, HCO at C6	Bending		2
	C, HC	1093	w	Heavy atom (CC, CO)	Stretching		2,5
	C, HC, L	1120	m	C, HC: Heavy atom (CC, CO); L: aromatic CH	C, HC: stretching; L: bending		2,5,6
	C	1151	sh	Heavy atom (CC, CO); HCC, HCO, HOC	CC, CO: stretching; HCC, HCO, HOC: bending		2,5
		1377	m	HCC, HCO, HOC	Bending		
		2740	w	?	?		2
	C, HC	2892	vs	CH, CH_2	Stretching		2,6
	L	1270	m	Aryl–O (aryl–OH), aryl–O–CH_3, guaiacyl/syringyl ring (C=O) mode	?		2,4,5
		1328		Aliphatic O–H	Bending		2,4
		1426	w	O–CH_3, CH_2, guaiacyl ring	O–CH_3: deformation; CH_2: scissoring; guaiacyl ring: vibration		2,5
		1459	m	O–CH_3, CH_2, guaiacyl ring	O–CH_3: deformation; CH_2: scissoring; guaiacyl ring: vibration		2
		1600	vs	Aryl ring	Symmetric stretching		2,4–6
		1663	m	Ring-conjugated C=C (coniferyl alcohol), C=O (coniferaldehyde)	Stretching		2,4,5
		2942	sh	CH_3, asymmetric CH (CH_3, O CH_3)			2,4–7
		3073	W	Aromatic C–H			2,4,6

ND-ac	C	381	m → w	Some heavy atom, CCC ring	Stretching; CCC: in-plane scissoring	±0	[2,3]
SE-ac							
OD-ac							
ND-ac	L	636	w → vw	Skeletons of aromatic rings, substituent groups and side chains	Deformation	+4	[2,4]
SE-ac			w				
OD-ac		640				+8	
ND-ac	C	911	vw → w	HCC, HCO at C6	Bending	+12	[2]
SE-ac		907				+8	
OD-ac		911				+12	
ND-ac	C, HC	1097	w	Heavy atom (CC, CO)	Stretching	+4	[2,5]
SE-ac							
OD-ac							
ND-ac	C, HC, L	1124	m	C, HC: Heavy atom (CC, CO); L: aromatic CH	C, HC: stretching; L: bending		[2,5,6]
SE-ac							
OD-ac							
ND-ac		1735	w	C=O	Stretching	–	[7]
SE-ac			m				
OD-ac		1739					
ND-ac		2942	sh	CH_3, asymmetric CH (CH_3, OCH_3)		±0	[2,4–7]
SE-ac							
OD-ac							
ND-ac	C	1151	sh	Heavy atom (CC, CO); HCC, HCO, HOC	CC, CO: stretching; HCC, HCO, HOC: bending		[2,5]
SE-ac							
OD-ac							
ND-ac		1339	m	HCC, HCO	Bending	–	
SE-ac							
OD-ac							
ND-ac		1381		HCC, HCO, HOC		+4	
SE-ac		1377				±0	
OD-ac		1381				+4	
ND-ac		2727	w	?	?	−13	[2]

SE-ac		2730				−10	
OD-ac							
ND-ac	C, HC	2899	vs	CH, CH_2	Stretching	+7	2,6
SE-ac							
OD-ac							
ND-ac	L	1274	m	Aryl–O (aryl–OH), aryl–O–CH_3, guaiacyl/syringyl ring (C=O) mode	?	+4	2,4,5
SE-ac							
OD-ac							
ND-ac		1328	m → nd	Aliphatic O–H	Bending	–	2,4
SE-ac							
OD-ac							
ND-ac		1426	w → nd	O–CH_3, CH_2, guaiacyl ring	O–CH_3: deformation; CH_2: scissoring; guaiacyl ring: vibration	–	2,5
SE-ac							
OD-ac							
ND-ac		1456	m			−3	2
SE-ac							
OD-ac							
ND-ac		1604	vs	Aryl ring	Symmetric stretching	+4	2,4–6
SE-ac							
OD-ac							
ND-ac		1663	m	Ring-conjugated C=C (coniferyl alcohol), C=O (coniferaldehyde)	Stretching	±0	2,4,5
SE-ac							
OD-ac							
ND-ac		3076	w	Aromatic C–H		+3	2,4,6
SE-ac		3069				−4	
OD-ac		3076				+3	

The 3421 cm^{-1} (s [b]) peak maximum corresponds to the O–H band for the non-acetylated case.[7] The 3415 (ND, SE), 3424 cm^{-1} (OD) [−6, +3 cm^{-1}, s → m] peak maximum corresponds to the O–H band.

[a] Sample: ac = acetylated.

[b] Component: C = cellulose, HC = hemicellulose, L = lignin; all components for softwood species.

[c] Strength: m = medium, nd = not detected, s = strong, sh = shoulder, vs = very strong, vw = very weak, w = weak.

[d] Type: type of the Raman response of the functionality/functionalities.

Table S2. Band assignments for the mean cell-corner Raman spectra of non-acetylated and acetylated sample sections.

Sample [a]	Component [b]	Raman shift (cm^{-1})	Strength [c]	Functionality	Type [d]	Change (cm^{-1})	Ref.
Non-ac	L	632	vw	Skeletons of aromatic rings, substituent groups and side chains	Deformation	–	2,4
	C, HC	1093	vw	Heavy atom (CC, CO)	Stretching		2,5
	C	1339	m	HCC, HCO	Bending		
	C, HC	2892	vw	CH, CH_2	Stretching		2,6
	L	1143	w	?	?		5
		1270	m	Aryl–O (aryl–OH), aryl–O–CH_3, guaiacyl/syringyl ring (C=O) mode			2,4,5
	C, HC, L	1452		C–H (CH_3, OCH_3)	Bending		6
	L	1600	vs	Aryl ring	Symmetric stretching		2,4–6
		1651	m	Ring-conjugated C=C (coniferyl alcohol), C=O (coniferaldehyde), C–C (coniferyl alcohol), C–O (coniferyl aldehyde)	Stretching		
		2942	s	CH_3, asymmetric CH (CH_3, O CH_3)			2,4–7
		3069	w	Aromatic C–H			2,4,6
ND-ac	L	640	vw	Skeletons of aromatic rings, substituent groups and side chains	Deformation	+8	2,4
SE-ac		636				+4	
OD-ac		640				+8	
ND-ac	C, HC	1097	vw	Heavy atom (CC, CO)	Stretching	+4	2,5
SE-ac		1093				±0	
OD-ac		1097				+4	
ND-ac	C	1339	m	HCC, HCO	Bending	±0	
SE-ac							
OD-ac							

ND-ac							
SE-ac	C, HC	2899	vw	CH, CH_2	Stretching	+7	2,6
OD-ac							
ND-ac		1132				−11	
SE-ac		1128	w	?		−15	5
OD-ac		1132				−11	
ND-ac	L			Aryl–O (aryl–OH), aryl–O–CH_3,	?		
SE-ac		1274		guaiacyl/syringyl ring (C=O)			2,4,5
OD-ac				mode			
ND-ac			m → w			+4	
SE-ac		1456		C–H (CH_3, OCH_3)	Bending		6
OD-ac							
ND-ac	C, HC, L	1735	vw				
SE-ac			w	C=O	Stretching	–	7
OD-ac		1739					
ND-ac							
SE-ac		1604	vs	Aryl ring	Symmetric stretching	+4	
OD-ac							
ND-ac		1663		Ring-conjugated C=C (coniferyl		+12	2,4–6
SE-ac			m	alcohol), C=O (coniferaldehyde),			
OD-ac	L	1666		C–C (coniferyl alcohol), C–O (coniferyl aldehyde)		+15	
ND-ac							
SE-ac		2942	s → vs	CH_3, asymmetric CH (CH_3, O CH_3)	Stretching	±0	2,4–7
OD-ac							
ND-ac							
SE-ac		3076	w	Aromatic C–H		+7	2,4,6
OD-ac							

The 3415 cm^{-1} (m ^{b}) peak maximum corresponds to the O–H band for the non-acetylated case.[7] The 3433 (ND), 3439 (SE), 3450 cm^{-1} (OD) [+18, +24, +35 cm^{-1}, m → vw] peak maximum corresponds to the O–H band.

[a] Sample: ac = acetylated.

[b] Component: C = cellulose, HC = hemicellulose, L = lignin; all components for softwood species.

[c] Strength: m = medium, nd = not detected, s = strong, sh = shoulder, vs = very strong, vw = very weak, w = weak.

[d] Type: type of the Raman response of the functionality/functionalities.

REFERENCES

(1) ÇeliK̇, A. E.; Can, A. Surface Characterization of Wood Treated with Acidic Deep Eutectic Solvents. *Eur. J. Wood Prod.* **2023**, *81* (1), 143–157. https://doi.org/10.1007/s00107-022-01843-1.

(2) Agarwal, U. P. Chapter 9: An Overview of Raman Spectroscopy as Applied to Lignocellulosic Materials. In *Advances in Lignocellulosics Characterization*; Argyropoulos, D. S., Ed.; TAPPI Press: Atlanta, GA, 1999; pp 201–225.

(3) Agarwal, U. P.; Ralph, S. A.; Baez, C.; Reiner, R. S. Detection and Quantitation of Cellulose II by Raman Spectroscopy. *Cellulose* **2021**, *28*, 9069–9079. https://doi.org/10.1007/s10570-021-04124-x.

(4) Agarwal, U. P.; McSweeny, J. D.; Ralph, S. A. FT–Raman Investigation of Milled-Wood Lignins: Softwood, Hardwood, and Chemically Modified Black Spruce Lignins. *J. Wood Chem. Technol.* **2011**, *31* (4), 324–344. https://doi.org/10.1080/02773813.2011.562338.

(5) Gierlinger, N.; Schwanninger, M. Chemical Imaging of Poplar Wood Cell Walls by Confocal Raman Microscopy. *Plant Physiol.* **2006**, *140* (4), 1246–1254. https://doi.org/10.1104/pp.105.066993.

(6) Mamleeva, N. A.; Kharlanov, A. N.; Kupreenko, S. Yu.; Chukhchin, D. G. Main Pathways of the Transformations of Lignocellulosic Material under the Action of Ozone. *Russ. J. Phys. Chem.* **2021**, *95* (11), 2214–2221. https://doi.org/10.1134/S0036024421110133.

(7) Ponzecchi, A.; Engelund Thybring, E.; Digaitis, R.; Fredriksson, M.; Solsona, S. P.; Thygesen, L. G. Raman Micro-Spectroscopy of Two Types of Acetylated Norway Spruce Wood at Controlled Relative Humidity. *Front. Plant Sci.* **2022**, *13*, 986578. https://doi.org/10.3389/fpls.2022.986578.
